# *Aspergillus niger* LBM 134 isolated from rotten wood and its potential cellulolytic ability

**DOI:** 10.1080/21501203.2020.1823509

**Published:** 2020-09-21

**Authors:** Gabriela Verónica Díaz, Romina Olga Coniglio, Clara Inés Chungara, Pedro Darío Zapata, Laura Lidia Villalba, María Isabel Fonseca

**Affiliations:** Laboratorio de Biotecnología Molecular, Instituto de Biotecnología Misiones “María Ebe Reca„ CONICET. Facultad de Ciencias Exactas, Químicas y Naturales. Universidad Nacional de Misiones. Ruta, Posadas, Misiones, Argentina

**Keywords:** *Nigri*, macromorphology, micromorphology, molecular markers, cellulases, fluorescence assays

## Abstract

Aspergillus

is a genus of filamentous and cosmopolitan fungi that includes important species for medical mycology, food, basic research and agro-industry areas. *Aspergillus* section *Nigri* are efficient producers of hydrolytic enzymes such as cellulases that are employed in the cellulose conversion. Hence, the search of new cellulolytic isolates and their correct identification is important for carrying out safe biotechnological processes. This study aimed to characterise the cellulolytic potential of *Aspergillus* sp. LBM 134, isolated from the Paranaense rainforest (Argentina) and to identify the isolate through a polyphasic approach. The fungus was identified as *Aspergillus niger* and its cellulolytic potential was evaluated by using Congo red technique and fluorescence plate assays for carboxymethyl cellulase, β-glucosidase and cellobiohydrolase, respectively. All three cellulase activities were positive; this bio-prospective positioned *A. niger* LBM 134 as a promising alternative for industries that require organisms capable of carrying out cellulosic biomass processing.

## Introduction

*Aspergillus* is a diverse group of filamentous and cosmopolitan fungi that grow in almost every natural and artificial substrate. These fungi have a significant impact in modern society and are very studied due to their importance in medical and industrial mycology (Samson et al. 2014; Park et al. 2017). Raper and Fennell (1965) recognised 150 species and currently, more than 340 species of *Aspergillus* are recognised in 4 subgenera and 19 sections (Houbraken et al. 2014; Samson et al. 2014; Park et al. 2017).

**Figure:**
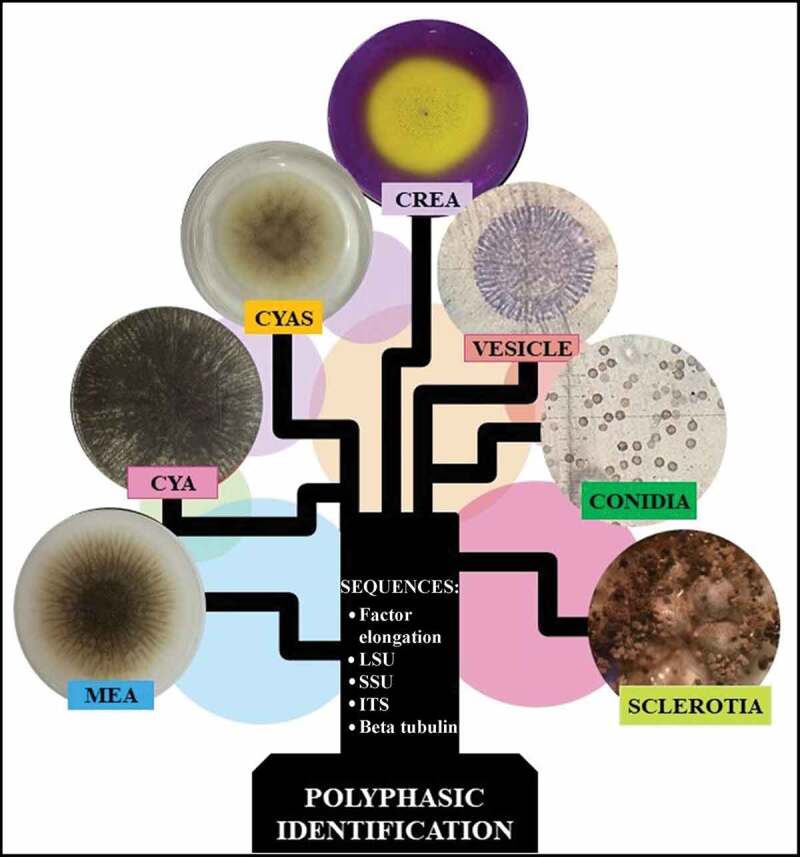

Black aspergillus is one of the most studied groups since they have huge industrial potential and biotechnological relevance (de Vries et al. 2017). They belong to subgenus *Circumdati* which comprises 26 species distributed into the clades *A. niger, A. carbonarius, A. heteromorphus, A. homomorphus* and *A. acualeatus* (Varga et al. 2011).

Previously, fungal identification was based almost entirely on macro- and micro-morphological criteria (Raper and Fennell 1965; Klich and Pitt 1988). However, identification of taxa in the genus *Aspergillus* is difficult (Abarca 2000; Samson et al. 2007a) and has now been complemented with molecular identification that compares specific genes or partial sequences named molecular markers (Bennett 2010). To identify *Aspergillus* species a multiple locus identification has been proposed that employs the use of ITS and also beta-tubulin (Bt), calmoduline (CMD) and transcriptional elongation factor 1α (Tef) molecular markers (Samson et al. 2014; Palumbo and O’keeffe 2015). Unfortunately, this multiple locus identification is often insufficient to identify species belonging to section *Nigri*. Therefore, a polyphasic approach has been recommended to delimit, identify and describe species of *Aspergillus* (Frisvad and Samson 2004; Samson et al. 2007b; Rodrigues et al. 2009; Simões et al. 2013; Silva et al. 2015; Decontardi et al. 2018). The polyphasic identification applies different types of data (DNA sequences, morphological, physiological and ecological data and extrolite analysis) in order to obtain a consensus result of higher fidelity and robustness. The polyphasic focus also avoids errors that arise when applying only morphological or/and molecular methods (Samson et al. 2004; Silva et al. 2011). Reference protocols of polyphasic identification have been proposed for different fungal groups adapting to more significant characters and several prestige laboratories offer this type of identification. The Westerdikj Fungal Biodiversity Institute (formerly CBS) has an interesting tool that is available free online, *Polyphasic Identification* for the identification of *Aspergillus, Penicillium* and yeast species comparing user data with its database, CBS-KNAW, to identify a fungal isolate through a polyphasic approach.

Species within *Aspergillus* section *Nigri* have been used in different industrial bioprocess with enzymatic production being one of the most popular applications of these fungi (Bennett 2010; Park et al. 2017). Cellulose degrading enzymes that attack cellulose have been intensely studied due to their wide industrial application (Martínez et al. 2005; Kuhad et al. 2011; Sohail et al. 2016; Park et al. 2017). The main cellulases are endo-β-1,4-glucanases (EG, EC 3.2.1.4), cellobiohydrolases (CBH, EC 3.2.1.91) and β-glucosidases (BGL, EC 3.2.1.21) (Singhania et al. 2017). EGs act on β-1,4-glucosidic bonds of cellulose to generate reducing and non-reducing ends that are attached by CBHI and CBHII, respectively, releasing cellobioses, which are then hydrolysed by BGLs (Singhania et al. 2017).

Cellulosic biomass processing is an attractive source of research that involves cellulases and cellulolytic organisms. Knowing more about new fungal isolates through a biotechnological focus would make available efficient enzymes for industry applications (Kuhad et al. 2011; Bertrand et al. 2017).

The present work addressed the identification of an *Aspergillus* isolate from Paranaense rainforest through a polyphasic approach, and investigated its biotechnological prospects by determining its cellulolytic potential.

## Materials and methods

### Isolate and morphological analysis

The isolate *Aspergillus* sp. LBM 134 belonging to the section *Nigri* was used in this study. It was selected from a previous study of screening and obtained from a sample of rotten wood in a natural zone of Misiones rainforest (Latitude 27°09ʹ05” S/Longitude 54°86ʹ77” W) during winter 2015 (Díaz et al. 2019a). The fungus was isolated by scrapping the material surface and inoculated on 39 g*L^−1^ potato dextrose agar (PDA) with 0.5% (p/v) chloramphenicol to inhibit bacterial growth (Benbow and Sugar 1999) and identified as *Aspergillus* sp. based on its macroscopic and microscopic characteristics using the keys of Carrillo (2003). The fungus was deposited in the Culture Collection of the Laboratory of Molecular Biotechnology (LBM) of the Institute of Biotechnology Misiones and was maintained on PDA medium at 4°C and incubated on the same medium at 28°C for its mycelial growth.

### DNA isolation, amplification and analyses

Total genomic DNA was extracted from axenic isolates grown for 3 d on 15 g*L^−1^ yeast extract 30 g*L^−1^ sucrose (YES) medium, following the protocols of Fonseca et al. (2015). Partial gene sequences were determined for the ITS1-5,8S gene-ITS2 region, using primers ITS1 and ITS4 (White et al. 1990), the large subunit 28S of rDNA gene (D1/D2) using primers NL1 and NL4 (Kurtzman and Robnett 1997), β-tubulin gene (Bt) using primers Bt2a and Bt2b (Glass and Donaldson 1995), calmodulin gene (CMD) using primers CMD5 and CMD6 (Hong et al. 2005) and the translation elongation factor 1-alpha gene (Tef) using primers EF1S and Tef1R (Yergeau et al. 2005; Samuels and Ismaiel 2009). Amplicons were sequenced in both directions and consensus sequences were determined using the software Chromas v2.6.5 and BioEdit Sequence Alignment Editor v7.0.5. Subsequent alignments were carried out using ITS, Bt and CMD sequences belonging to type strains (less *Aspergillus* sp. LBM 134) of culture collection CBS-KNAW referenced by Varga et al. (2011). Alignments were generated for each individual locus and then assembled using MEGA v.7.0 and manually corrected if necessary. Concatenated sequences were included in the phylogenetic inference based on maximum likelihood (ML) and neighbour joining (NJ) applying substitution Kimura’s model and a bootstrap with 1 000 replicates. *A. flavus* CBS 100927 was used as external group. The phylogenetic inference was carried out with software MEGA version 7.0.

### Polyphasic identification

Polyphasic identification was carried out through the online tool Polyphasic Identification of the website http://www.westerdijkinstitute.nl/Aspergillus/DefaultInfo.aspx?Page=Home of the Westerdijk Fungal Biodiversity Institute of Royal Netherlands Academy of Arts and Sciences. Polyphasic identification required the following information of *Aspergillus* sp. LBM 134: macro-morphology, micro-morphology, acid production tests and DNA sequences of ITS, D1/D2, Bt, CMD and Tef.

#### Macro-morphology

*Aspergillus* sp. LBM 134 was grown on different media as recommended by the International Commission for *Penicillium* and *Aspergillus* for polyphasic identification. *Aspergillus* sp. LBM 134 growth was measured and macro-morphological characteristics were observed. The culture media were Czapek yeast extract agar (CYA; 3 g*L^−1^ NaNO_3_, 5 g*L^−1^ yeast extract, 30 g*L^−1^ sucrose, 1.3 g*L^−1^ K_2_HPO_4_.3H_2_O, 0.5 g*L^−1^ MgSO_4_.7H_2_O, 0.5 g*L^−1^ KCl, 0.01 g*L^−1^ FeSO_4_.7H_2_O and 15 g*L^−1^ agar, pH 6.3 ± 0.2 (Pitt 1979); malt extract agar (MEA; 30 g*L^−1^ malt extract, 1 g*L^−1^ bacteriological peptone, 20 g*L^−1^ glucose and 20 g*L^−1^ agar, pH 5.3 ± 0.3) and, Czapek Yeast Extract with salt (CYAS; 3 g*L^−1^ NaNO_3_, 50 g*L^−1^ NaCl, 5 g*L^−1^ yeast extract, 30 g*L^−1^ sucrose, 1.3 g*L^−1^ K_2_HPO_4_.3H_2_O, 0.5 g*L^−1^ MgSO_4_.7H_2_O, 0.5 g*L^−1^ KCl, 0.01 g*L^−1^ FeSO_4_.7H_2_O and 15 g*L^−1^ agar, pH 6.3 ± 0.2) (Pitt 1979). All media were autoclaved at 121°C for 20 min to sterilise. *Aspergillus* sp. LBM 134 was inoculated in the centre of 90 mm Petri plate containing the media outlined above and incubated at 25°C in the dark. The fungus was also incubated at 30°C and 37°C in CYA medium. Cultures were examined each day and measured at 7 d of incubation.

#### Micro-morphology

*Aspergillus* sp. LBM 134 was grown on MEA and PDA media at 28°C for 3 d to observe microscopic characteristics of mycelium. Young mycelium was stained by cotton blue-lactophenol technique. Micro-characteristics of stipes, conidiophores, vesicles, phialides, conidia, cleiostothecia/sclerotia, ascospores were photographed by a digital Canon Power Shot camera G10 and measured using the software *ImageJ*.

#### Growth and acid production on creatine sucrose agar (CREA) culture medium

The capability of growth and production of acid by the fungus was tested on CREA medium: 3.0 g*L^−1^ creatine, 30 g*L^−1^ sucrose, 0.5 g*L^−1^ KCl, 0.5 g*L^−1^ MgSO_4_.7H_2_O, 0.5 g*L^−1^ FeSO_4_.7H_2_O, 1.3 g*L^−1^ K_2_HPO_4_.3H_2_O, 0.05 g*L^−1^ Bromocresol purple and 15.0 g*L^−1^ agar (Frivsad 1985). The pH of the medium was adjusted to 8 with Tris buffer and autoclaved at 121°C for 20. The fungus was inoculated at the centre of Petri plates containing CREA medium and incubated at 25°C in the dark for 7 d. If violet CREA medium turns yellow after incubation, then the fungus produces acids on this medium.

#### Test of Ehrlich to evaluate the cyclopiazonic acid production

*Aspergillus* sp. LBM 134 was examined for the production of cyclopiazonic acid and other alkaloids reacting with Ehrlich reagent (2 g 4-dimethylaminobenzaldehyde in 85 mL of 96% ethanol and 15 mL of 10 N HCl) (Lund 1995) using the filter paper method. Firstly, *Aspergillus* sp. LBM 134 was grown on CYA medium and incubated at 25°C for 7 d. Plugs of 5 mm diameter covered with fungal mycelium were cut out of the centre of the colony and a 1 cm x 1 cm square of filter paper (Whatman No. 1) wetted with Ehrlich reagent was placed on the mycelial side of the plug. The presence of violet, yellow or pink/red ring around the plug after 2 to 6 min, means that the fungus produces cyclopiazonic acid or related alkaloids (Frisvad and Samson 2004).

### Qualitative determination of cellulolytic potential Aspergillus sp. LBM 134

#### Determination of CMCase activity

CMCase activity of *Aspergillus* sp. LBM 134 was carried out on agar plates containing Czapek-agar medium (2 g*L^−1^ NaNO_3,_ 1 g*L^−1^ K_2_HPO_4_, 0.5 g*L^−1^ KCl, 0.5 g*L^−1^ MgSO_4_.7H_2_O, 0.01 g*L^−1^ FeSO_4_.7H_2_O, 20 g*L^−1^ agar) supplemented with 0.1% CMC. The pH of the medium was adjusted to 4.5 ± 0.2 with 100% glacial acetic acid. Plugs (5 mm diameter) were placed in the centre of Petri dishes containing the medium. A non-inoculated plate and an inoculated medium without the substrate (CMC) were used as negative controls. After incubation at 28°C for 4 d, the colony diameter was measured as growth halo and all Petri dishes were incubated at 50°C for 60 min covered with 50 mM sodium acetate buffer at pH 4.8 ± 0.2. Then, plates were stained with an aqueous solution of 0.1% Congo red and shaken at 80 rpm for 15 min. The Congo red solution was then poured off by washing with distillated water and 1 M NaCl solution to reveal the degradation halo which was measured. The cellulose degradation coefficient (CDC) was determined as follows:

$$CDC=dh*d_{g-1}$$

where dh is the degradation halo, dc is the colony diameter.

#### Determination of BGL and CBH activities of Aspergillus sp. LBM 134 by fluorescence plate assays

The fungus was inoculated on agar plates containing 1 g*L^−1^ CMC, 1 g*L^−1^ yeast extract and 20 g*L^−1^ agar supplemented with 0.1 mM 4-methyl umbelliferyl glucoside (Mu-g; Sigma, MO, USA) for BGL determination and 0.1 mM 4-methyl umbelliferyl cellobioside (Mu-c; Sigma, MO, USA) for CBH assay. The pH of the media was adjusted to 4.2 with 100% glacial acetic acid. Non-inoculated plates and inoculated plates without the substrates were used as negative controls. All plates were incubated at 28 ± 2°C for 3 d. After incubation, the plates were exposed under UV light. If the fungus shows a fluorescence halo under UV light, it is considered as positive BGL or CBH producer.

### Quantitative determination of Aspergillus sp. LBM 134 cellulolytic potential

*Aspergillus* sp. LBM 134 was grown according to Díaz et al. (2019b). After incubation, the culture media were centrifuged at 4°C (15 min, 10,000 g) and supernatants were used to determine CMCase, BGL, CBH and Filter Paper activity (FPase).

CMCase and FPase activities were determined according to Ghose (1987) using CMC (Sigma-Aldrich, USA) and Whatman no. 1 filter paper as substrates, respectively. Reducing sugars were measured by 1,3-dinitrosalicylic acid (DNS) assay (Miller 1959) using glucose as standard curve. Enzyme activities were expressed as international units (U), defined as the amount of enzyme needed to produce 1 µmol of glucose per min at 50°C.

BGL activity was assayed by the method described by Herr et al. (1978) using nitrophenyl-β-D-glucoside (pNPG; Sigma, MO, USA) as substrate. The CBH activity was assayed following a modified method described by Wu et al. (2006) using nitrophenyl-β-D-cellobioside (pNPC; Sigma, MO, USA) as substrate. In both enzyme assays, the amount of p-nitrophenol released was measured and expressed as international units (U), defined as the amount of enzyme necessary to release 1 μmol of p-nitrophenol per minute at 50°C.

## Results

### Concatenated sequences trees

Using only ITS sequences, the trees formed did not distinguish *Aspergillus* sp. LBM 134 from *A. lacticoffetaus, A. welwitschiae* (*A. awamori* sensu Perrone), *A.foetidus* and *A. niger* (subclade *A. niger*) (data not shown). Therefore, in this work, we amplified for ITS, Bt, CMD and also for D1-D2 and Tef. The consensus sequences are deposited in GenBank of National Centre for Biotechnology Information (NCBI). The sequence access numbers are MK457457, MK465342, MK465341, MK463630 and MK465340, respectively.

**Figure 1.**
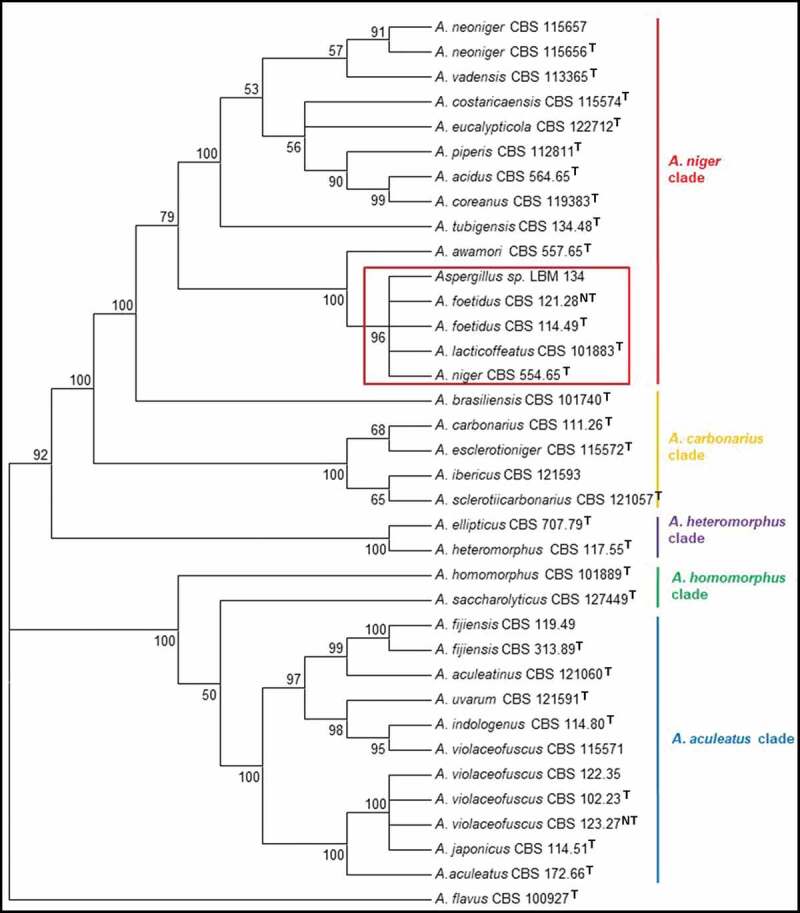
Phylogenetic tree obtained by the neighbour joining method, showing placement of *Aspergillus* sp. LBM 134 among species of section *Nigri* and reference species *A. flavus* (the outgroup species in the analysis) as presented by the bootstrap consensus tree inferred from 1 000 replicates derived from analysis of ITS-Bt-CMD. The percentages of replicate trees in which the associated taxa clustered together in the bootstrap test (1 000 replicates) are shown next to the branches. The tree is drawn to scale, with branch lengths in the same units as used for evolutionary distances used to infer the phylogenetic tree. The evolutionary distances were computed using Kimura’s method. All but LBM 134 are type strains

**Figure 2.**
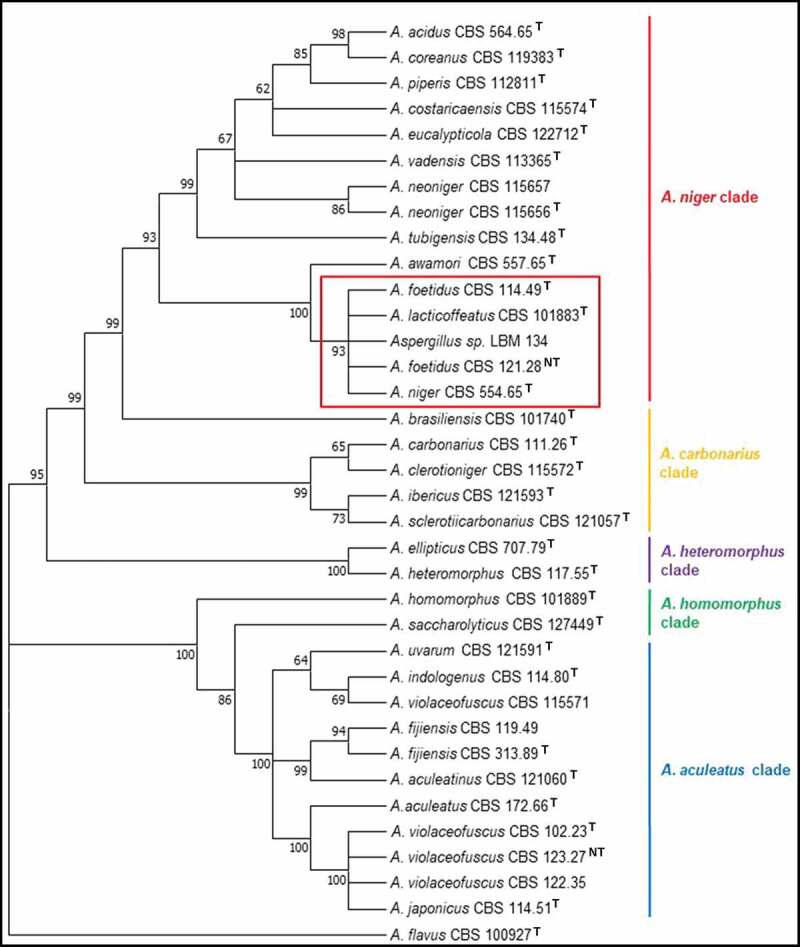
Phylogenetic tree obtained by the maximum likelihood method, showing placement of *Aspergillus* sp. LBM 134 among species of section *Nigri* and reference species *A. flavus* (the outgroup species in the analysis) as presented by the bootstrap consensus tree inferred from 1 000 replicates derived from analysis of ITS-Bt-CMD. The percentages of replicate trees in which the associated taxa clustered together in the bootstrap test (1 000 replicates) are shown next to the branches. The evolutionary distances were computed using Kimura’s method. All but LBM 134 are type strains

ITS, Bt and CMD sequences were used and they were concatenated for constructing NJ and ML trees. The alignment of the concatenated sequences was deposited in the website TreeBASE (https://treebase.org/) under the submission number: TB2:S24742 (http://purl.org/phylo/treebase/phylows/study/TB2:S24742). These trees grouped the CBS type strains into the expected clades of section *Nigri: A. niger, A. carbonarius, A. heteromorphus, A. homomorphus, A.aculeatus*. The phylogenetic tree using NJ method showed *Aspergillus* sp. LBM 134 belonged to the subclade *A. niger* and grouped with *A. niger* CBS 554.65, *A. lacticoffeatus* CBS 101883, *A. foetidus* CBS 114.49 and *A. foetidus* CBS 121.28, with a bootstrap value of 96 (Figure 1). The tree also showed that the subclade *A. niger* was included into the clade *A. niger* with the other subclade *A. tubigensis* with the following type species: *A. piperis, A. vadensis, A. costaricaencis, A. acidus* and *A. neoniger*. Similarly, the tree obtained by ML method grouped *Aspergillus* sp. LBM 134 with *A. niger* CBS 554.65, *A. lacticoffeatus* CBS 101883, *A. foetidus* CBS 114.49 and *A. foetidus* CBS 121.28, with a bootstrap value of 93 (Figure 2) staying inside the subclade *A. niger* and the clade *A. niger*. Moreover, the analysis using concatenated sequences could set aside *A. awamori* from the cluster formed using only ITS sequences. Therefore, we discarded that the isolate was *A. welwitschiae*. However, due to the impossibility to appropriately identify our isolate and reach to a species-specific identification, we carried out a polyphasic identification.

### Polyphasic identification

**Figure 3.**
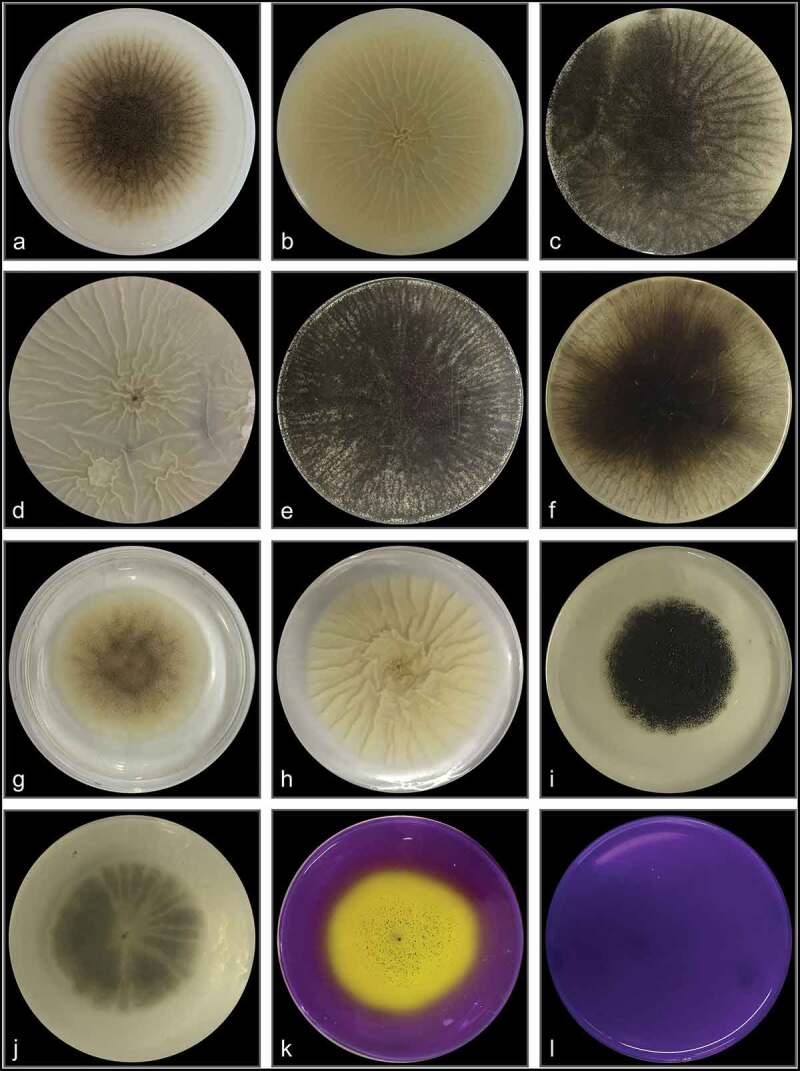
Macro-morphology and production of acid-base of *Aspergillus* sp. LBM 134 grown for 7 d on different agar media. On MEA at 25 C: (**a**) conidia colour and (**b**) reverse colour; CYA at 25 C: (**c**) conidia colour and (**d**) reverse colour; CYA at 30 C: (**e**) conidia colour and (**f**) reverse colour; CYA at 37 C: (**g**) conidia colour and (**h**) reverse colour; CYAS at 25 C: (**i**) conidia colour and (**j**) reverse colour. Acid production on CREA medium: (**k**) inoculated plate and (**l**) plate without inoculating

*Aspergillus* sp. LBM 134 showed differences in its macro-morphology in different media and temperatures (Figure 3). The colour of the “top view” of the mycelium is due to the fungus conidia colour. Black colouration, characteristic for conidia of black *Aspergillus*, and cream reverse were observed on MEA medium (Figure 3(a-b)). The conidia were greenish browns to dark brown or almost black on CYA media, getting darker as the temperature of incubation increased (from 25°C to 37°C); the colour of the reverse also showed the same pattern, from cream to dark brown as the temperature of incubation increased (Figure 3(c-h)). Conidia of paler colour were produced on CYAS medium as well as the colour reverse (Figure 3(i-j)). *Aspergillus* sp. LBM 134 grown on MEA or CYAS media did not cover all the plate during the incubation time. Data are summarised in Table 1.

**Table 1.** Macro-morphological characteristics registered for *Aspergillus* sp. LBM 134 grown on different media for 7 d of incubation

Culture media	Incubation temperature (°C)	Colony diameter (mm)	Conidia colour	Reverse colour
MEA	25	51–63	black	white to cream
	25	90	olive	white to cream
CYA	30	90	brown to dark brown	olive to soft brown
	37	90	dark brown to black	dark brown
CYAS	25	60–61	olive to soft brown	cream

**Figure 4.**
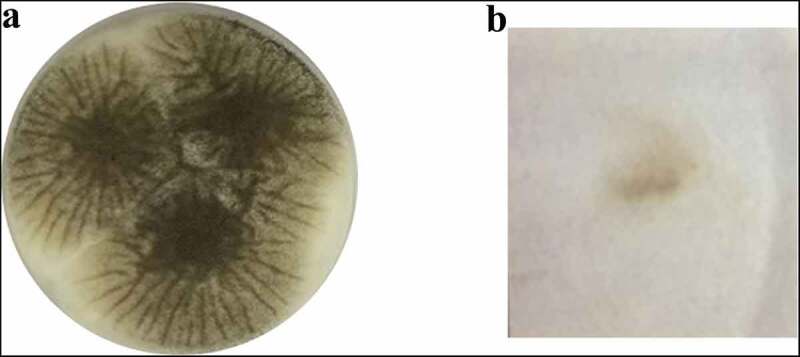
Test of Ehrlich reagent using the filter paper method to detect cyclopiazonic acid and related alkaloids. (**a**) *Aspergillus* sp. LBM 134 developed on CYA medium where plugs were cut off. (**b**) Filter paper did not show any colour ring indicating the absence of production of cyclopiazonic acid or any related alkaloid

*Aspergillus* sp. LBM 134 grown on CREA medium registered a moderate production of acids visualised by the turn of violet to yellow of culture medium (Figure 3(k-l)). Positive acid production on CREA medium is reported for both *A. niger* and *A. lacticoffeatus*. The same occurs for the Ehrlich test; the Ehrlich reagent did not react with the *Aspergillus* sp. LBM 134 mycelium (Figure 4) suggesting that it does not produce cyclopiazonic acid or any related alkaloid.

**Figure 5.**
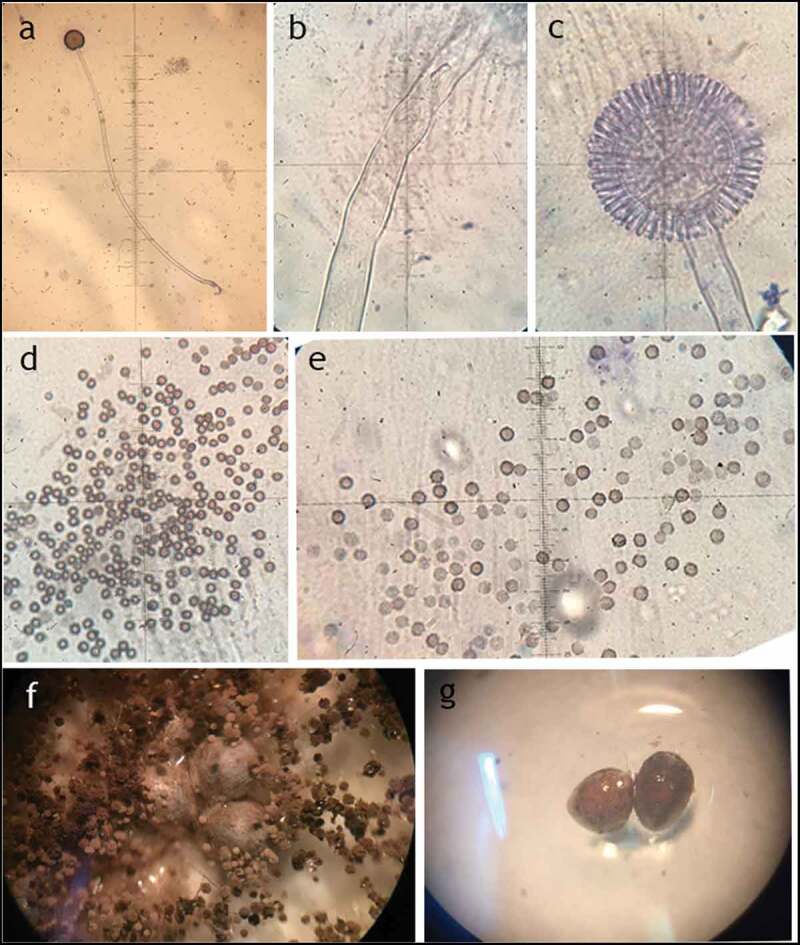
Micro-morphology of *Aspergillus* sp. LBM 134. (**a**) Conidiophore, 10X; (**b**) stipe, 400X; (**c**) vesicle, 400X; (**d**) and (**e**) conidia, 1000X; (**f**) and (**g**) sclerotia, 4X

Micro-morphological characteristics of *Aspergillus* sp. LBM 134 are shown in Table 2 and Figure 5. Stipes were wide, hyaline, long without any ornamentation (Figure 5(a-b)). *A. niger* has hyaline conidiophores with long and hyaline stipes, similar to *Aspergillus* sp. LBM 134; stipes from *A. lacticoffeatus* are shorter and orange. Vesicles of *Aspergillus* sp. LBM 134 had almost a spherical shape and metulae (Figure 5(c)) and conidia have apiculate ornamentation (Figure 5(d-e)). An interesting characteristic shown by *Aspergillus* sp. LBM 134 grown on CYA medium for 14 d was the presence of sclerotia (Figure 5(f-g)). Sclerotia are asexual, multicellular reproduction structures in fungi (Chet and Henis 1975) and there is no study that describes *A. lacticoffeatus* species as sclerotia producer.

**Table 2.** Micro-morphological characteristics of *Aspergillus* sp. LBM 134 grown on CYA and MEA media for 3 d of incubation

Structure	Length (µm)	Width (µm)	Shape and ornamentation
Stipe	1 000.0 ± 300.0	15.0 ± 5.0	-
Vesicle	39.0 ± 1.0	40.0 ± 1.0	biseriate; globose to sub-globose
Phialides	-	-	cylindric
Conidia	3.0 ± 0.5	2.6 ± 0.5	sub-globose to ellipsoidal; thick walls; finely echinulate

After analysing all this information (macro, micro-morphological, acid and alkaloid production) about *Aspergillus* sp. LBM 134 and the sequence information (ITS, D1-D2, Bt, CMD and Tef) we introduced this information into the online tool Polyphasic Identification and identified the isolate LBM 134 as *A. phoenicis*, currently, *A. niger.*

### Cellulolytic bioprospection of A. niger LBM 134

**Figure 6.**
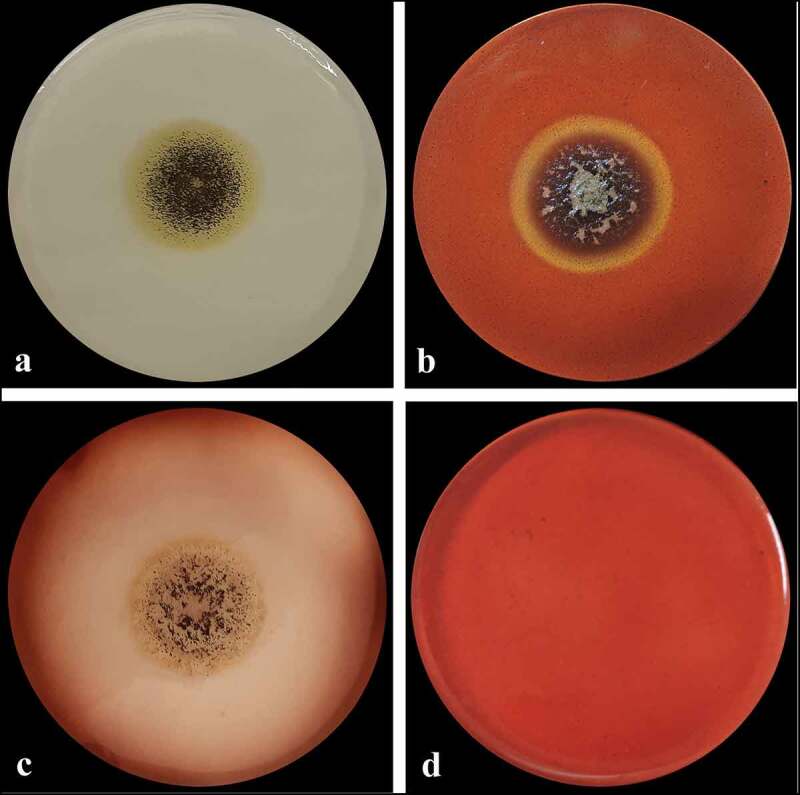
CMCase activity of *Aspergillus* sp. LBM 134 determined by the Congo red assay. (**a**) Developed mycelium for 4 d without revealing; (**b**) developed mycelium on medium with the substrate (CMC) and revealed showing a degraded halo around the mycelium; (**c**) control plate, developed mycelium on medium without substrate; (**d**) plate control, medium without inoculating

For CMCase activity determination, *A. niger* LBM 134 was grown on Czapek medium supplemented with CMC for 4 d (Figure 6(a-d)). Degradation of CMC in agar-plates, which may be considered a semiquantitative technique, revealed that *A. niger* LBM 134 had a value of CDC of 1.2 ± 0.01, indicating that the degradation halo is greater than the growth halo.

**Figure 7.**
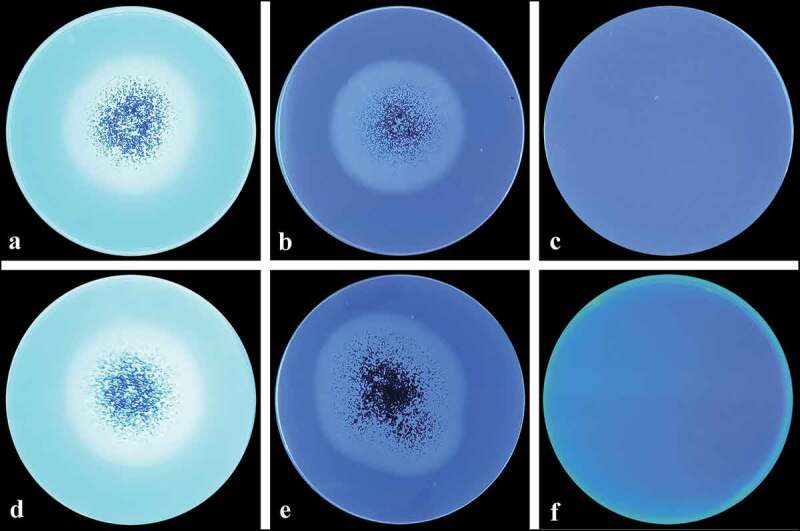
BGL and CBH activities of *Aspergillus* sp. LBM 134 determined by fluorescence plate assay. (**a**) Developed mycelium for 4 d on medium containing the substrate (Mu-g) and revealed; (**b**) plate control, developed mycelium for 4 d on medium without the substrate; (**c**) control plate, medium containing the substrate without inoculating. (**d**) Developed mycelium for 4 d on medium containing the substrate (Mu-c) and revealed; (**e**) plate control, developed mycelium for 4 d on medium without the substrate; (**f**) control plate, medium containing the substrate without inoculating

On the other hand, screening for BGL and CBH enzymes in agar plates may be considered exclusively qualitative and *A. niger* LBM 134 was classified as positive (Figure 7). Furthermore, EG, BGL, CBH and PFase were quantitatively determined in two optimised media (Table 3) showing high levels on these enzymatic enzymes.

**Table 3.** CMCase, BGL, CBH and FPase activities of *Aspergillus* niger LBM 134 grown on two optimised media supplemented with sugarcane bagasse (SCB) and cassava bagasse (CB)

Enzyme activities (UmL^−1^)	SCB	CB
**CMCase**	2.72 ± 0.23 UmL^−1^	3.72 ± 0.25 UmL^−1^
**BGL**	0.29 ± 0.00 UmL^−1^	0.43 ± 0.00 UmL^−1^
**CBH**	0.17 ± 0.00 UmL^−1^	0.28 ± 0.00 UmL^−1^
**FPase**	0.35 ± 0.00 UmL^−1^	0.38 ± 0.00 UmL^−1^

## Discussion

The taxonomy of filamentous fungi is complicated and classifying the species of *Aspergillus* is not an exception (Samson et al. 2007a). Identifying *Aspergillus* species of section *Nigri* is not easy due to their phenotypic similarities (macro and microscopic) and therefore, it is necessary to use several strategies for identifying them. DNA sequence information is increasingly being used for species identification and diagnosis. Although ITS is known as the fungal barcode (Schoch et al. 2012), this molecular marker does not allow to identify closely related species. Some authors (2017; Samson et al. 2014) recommended the use of a second or even a third molecular marker for identifying black aspergillus. In this sense, the use of Bt, CMD, RPB2 and Tef gene sequences in addition of ITS would achieve a species-specific identification.

While RPB2 is not easy to amplify, Tef is not reported for all *Aspergillus* species in the database. Bt is the easiest gene to amplify and almost all species of section *Nigri* can be identified using this gene sequence although it is impossible to discriminate between Bt sequences belonging to *A. niger* and *A. lacticoffeatus*. Moreover, there is a risk of amplifying Bt paralog genes (Koonin 2005) because of the variations inside the gene introns and their number (Peterson 2008; Hubka and Kolarik 2012; Samson et al. 2014). The same phenomenon occurs with CMD gene; it is easy to amplify and has the capacity to distinguish most species of *Aspergillus*; so is the second temporal marker proposed for *Aspergillus* (Samson et al. 2014).

In this work, the trees formed using the NJ and ML methods grouped the type species CBS into the expected clades of section *Nigri*, reproducing the phylogeny for the fungi group described by Varga et al. (2011) and Samson et al. (2014). Both trees showed that *Aspergillus* sp. LBM 134 belonged to the subclade *A. niger*. The use of international renowned species such as CBS type strains ensures that the trees were constructed with correct sequences and taxonomic names (Fungaro et al. 2017). We used that criteria and decided to not use any sequence submitted in GenBank since it is a public, archival database and accepts all sequences submitted and cannot always verify the taxonomy. Hence, results from BLAST search may give hits to misidentified sequences in the database (Fungaro et al. 2017).

*A. niger* LBM 134 has been grouped within the subclade *A. niger* with the species *A. welwitschiae, A. foetidus* and *A. lacticoffeatus* by using only molecular techniques. The trees using concatenated sequences separated *A. welwitschiae* from the *A. niger* clade. For a long time, *A. welwitschiae* was considered as a synonym of *A. niger*. Currently, both species are considered as cryptic species (Perrone et al. 2011) in speciation process; hence, it is necessary to use more than one molecular marker to distinguish them (Samson et al. 2014; von Hertwig et al. 2018). Regarding *A. foetidus* is not currently use anymore and the correct name is *A. niger* (Samson et al. 2004; Varga et al. 2011). *A. lacticoffeatus* was reported for the first time in 2004 by Frisvad and Samson (2004), is in the list of accepted species of genus *Aspergillus*. However, taxonomic studies by Varga et al. (2011), de Vries et al. (2017) and Vesth et al. (2018) and the study of growth and hydrolytic profiles by Meijer et al. (2011) considered the species *A. lacticoffeatus* as a synonym of *A. niger*. A study of *A. lacticoffeatus* strains isolated from coffee grains in Venezuela and Indonesia (Frisvad and Samson 2004) suggested that *A. lacticoffeatus* could be a mutant strain of *A. niger*.

A polyphasic approach was used to distinguish if the isolate LBM 134 was *A. niger or A. lacticoffeatus* and LBM 134 was correctly identified as *A. niger*. The colour of conidia (greenish brown-black) developed by *A. niger* LBM 134 on MEA and CYA which effectively coincides with conidia from *A. niger* (Abarca 2000) and contrasts with soft brown conidia from *A. lacticoffeatus* (Samson et al. 2004; Cabañes and Bragulat 2018). Another characteristic feature was the ornamentation of conidia of *A. niger* LBM 134; these had their surface less echinulate than *A. lacticoffeatus* conidia (Frisvad and Samson 2004). Also, *A. niger* LBM 134 showed sclerotia production, characteristic for *A. niger* grown on CYA medium supplemented with fruit pieces and rice as reported by Frisvad et al. (2014). The Polyphasic identification online tool showed that the isolate LBM 134 was *A. phoenicis*, this name is antique and correspond to *A. niger* (as we call it henceforth) (Abarca et al. 2004). The employment of online software to identify species allows to verify the obtained results in the laboratory and, at the same time, eliminates the subjectivity when putting weight on the criteria used in identification. Although the software solved that the isolate LBM 134 was *A. phoenisis*, this denomination is rejected over the conserved species *A. niger* (Frisvad et al. 1990; Kozakiewicz et al. 1992). This fact emphasises the importance of a critical analysis carried out by the researcher on the available bioinformatic tools.

Black *Aspergillus* are efficient producers of hydrolytic enzymes and *A. niger* has been employed for enzyme production to be used in different biotechnological processes. Among hydrolytic enzymes, we focused on cellulase production by *A. niger* LBM 134 since they are essential enzymes for the bioprocessing of cellulosic biomass. As *A. niger* LBM 134 was isolated from rotten wood, we understood that this fungus was a potential cellulolytic producer due to it grew on cellulosic biomass and probably used cellulose as carbon and energy. For carrying out the bio-prospective studies of *A. niger* LBM 134, we employed qualitative methods for detection of cellulase enzymes. In first place, we evaluated EG activity on plate assay using CMC as substrate using Congo red, a colourant that interacts with β-1,4-glucosidic bonds (Sazci et al. 1986), contrasting with the discoloured halo that represents the degraded substrate by CMCase activity. We measured the CMCase activity by determining the CDC which is the most used parameter for evaluating the cellulase production by microorganisms on solid media. There is a direct correlation between the degradative diameter and the degradation capacity (Lin et al. 1991). Since CMC can be degraded by other enzymes in addition to EG, talking about CMCase activity is correct. In this sense, we can say that *A. niger* LBM 134 has positive CMCase activity which coincides with the literature (Castrillo et al. 2012; Martínez et al. 2005; Panda et al. 2012; Singhania et al. 2017; Maia and Fraga 2017). Respect on BGL and CBH activities, the fluorescence was intense after a few days of incubation; data agreed with the literature that describes species of *A. niger* as BGLs and CBHs producer and the technique of fluorescence plate contributed more significant results in the study of the potential cellulolytic of *A. niger* LBM 134. These qualitative, novelty, specific and simple assays provide key information when investigating new isolates not yet characterised that could have a big biotechnological potential, for example, in the cellulosic bioconversion (Coniglio et al. 2017). To our knowledge, this is the first time the substrates Mu-g and Mu-c were used for qualitative determination of BGL and CBH activities in ascomycetes.

Cellulolytic potential of *A. niger* LBM 134 was reinforced by quantitative determination of CMCase, BGL, CBH and PFase. Levels of these cellulolytic enzymes of *A. niger* LBM 134 were higher than values found in other species of *Aspergillus* grown on CMC (El-Hadi et al. 2014; Sohail et al. 2016). Furthermore, the cellulolytic ability of *A. niger* LBM 134 was confirmed by the successful bioconversion of two agroindustrial wastes: sugarcane bagasse and cassava bagasse (paper in press).
